# Performance in Sound-Symbol Learning Predicts Reading Performance 3 Years Later

**DOI:** 10.3389/fpsyg.2018.01716

**Published:** 2018-09-12

**Authors:** Josefine Horbach, Kathrin Weber, Felicitas Opolony, Wolfgang Scharke, Ralph Radach, Stefan Heim, Thomas Günther

**Affiliations:** ^1^Child Neuropsychology Section, Department of Child and Adolescent Psychiatry, Psychotherapy and Psychosomatics, Medical School, RWTH Aachen University, Aachen, Germany; ^2^Department of Neurology, Medical Faculty, RWTH Aachen University, Aachen, Germany; ^3^Allgemeine und Biologische Psychologie, Bergische Universität Wuppertal, Wuppertal, Germany; ^4^Department of Psychiatry, Psychotherapy and Psychosomatics, Medical Faculty, RWTH Aachen University, Aachen, Germany; ^5^Research Centre Jülich, Institute of Neuroscience and Medicine (INM-1), Jülich, Germany; ^6^Faculty of Health, Zuyd University, Heerlen, Netherlands

**Keywords:** predictors of reading, sound-symbol learning, longitudinal study, letter knowledge, dynamic test

## Abstract

To master the task of reading, children need to acquire a coding system representing speech as a sequence of visual symbols. Recent research suggested that performance in the processing of artificial script that relies on the association of sound and symbol may be associated with reading skill. The current longitudinal study examined the predictive value of a preschool sound-symbol paradigm (SSP) of reading performance 3 years later. The Morse-like SSP, IQ, and letter knowledge (LK) was assessed in young preschool children. Reading outcome measures were examined 3 years later. Word reading, pseudoword reading, and reading comprehension were predicted with age, IQ, LK, and SSP. The results showed that SSP substantially predicted reading fluency and reading comprehension 3 years later. For reading fluency measures, the influence of further predictor variables was not significant and SSP served as a sole predictor. Reading comprehension was best explained by SSP and age. The amount of variance SSP explained in reading 3 years later was remarkably high, with an explained variance between 63 and 82%, depending on the outcome reading variable. SSP turned out to be a substantial predictor of later reading performance in a language with statistically reliable spelling-to-sound relations. As LK is highly dependent on educational support, we assume that children in our socioeconomically diverse sample did not have much opportunity to acquire LK in their home environment. In contrast, the SSP challenges students to acquire new spelling-to-sound relations, simulating a core aspect of natural reading acquisition. Future work will test this paradigm in less transparent languages like English and explore its potential as a future standard assessment in the study of early reading development.

## Introduction

The current longitudinal study investigates the predictive value of the performance of preschoolers in a sound-symbol paradigm (SSP) on later reading achievement. The paradigm is based on an earlier study of Horbach et al. (2015) which found SSP to better predict later reading in six-year-old monolingual kindergarteners over and above the established predictors phonological awareness (PA), rapid automatized naming (RAN), short-term memory (STM), and environmental factors. Due to the simplistic design of SSP, our study is able to assess SSP’s predictive capacity for younger preschool children.

The ability to read is crucial for participation in our society. Reading difficulties start early in childhood and tend to persist throughout reading development (Cunningham and Stanovich, 1997; Landerl and Wimmer, 2008). Such difficulties can substantially limit academic performance and career choices (Esser et al., 2002). Therefore, it is important to improve diagnostic tools in order to identify and prevent risk for reading difficulties in children as early as possible.

To master the task of reading, children need to acquire a coding system representing speech as a sequence of visual symbols (Ziegler and Goswami, 2005). Necessary processes for this are first, to learn the association between sound and symbol and second, to serially process the learned correspondences. Recent studies examining the role of sound-symbol learning in reading have used paradigms that require the serial processing of newly-learned visual–verbal correspondences and assessed the relation of performance on these tasks with reading ability. Aravena et al. (2013) developed an artificial orthography and demonstrated that normal readers performed better than students with dyslexia in serial application of the newly-learned sound-symbol associations. Interestingly, normal readers differed from dyslexic readers in *serial* processing of new letter names, even though they did not differ in their knowledge of the new letter names themselves. In a further study, the authors showed that a 20 min training on the artificial orthography was enough to differentiate dyslexic from non-dyslexic readers (Aravena et al., 2017).

Participants in the studies of Aravena et al. (2013, 2017) already had several years of reading experience when tested. In a study of Horbach et al. (2015), the predictive power of a Morse-like SSP was assessed in monolingual kindergarteners without the experience of formal reading instruction. This task was designed to simulate the process of learning to read schematically. First, children learned to associate verbal sounds with graphical symbols, similar to a classical paired-associate learning task. Afterward, children had to recall strings of the newly-learned correspondences, similar to Morse-code. The children learned only two associations to keep the influence of phonological processing and working memory load as low as possible. The authors found that SSP predicted word reading one year later in non-readers over and above PA, verbal STM, and RAN. A group of children were able to read before they received formal reading instruction. In these early readers, SSP did not predict reading in first grade but so did early reading performance measured in kindergarten. It was concluded that SSP simulates the process of learning to read and is therefore especially appropriate for young *preliterate* children. Gellert and Elbro (2017) replicated these findings using a similar paradigm of artificial decoding in kindergarten children for the prediction of reading in the first grade. The children had to learn three sound-symbol pairs and blend them into new words. Their study found the test predicted reading significantly after controlling for several standard predictors. The authors suggested the *learning aspect* of the task is essential for the prediction of initial reading development.

Some years before, the authors demonstrated a further advantage of SSPs in the prediction of reading (Elbro et al., 2012); the measurement is language independent. From a global perspective, multilingualism is normality (Riehl, 2014). In 2016, 38% of children under an age of 10 had a migration background in Germany (Statistisches Bundesamt, 2017). Therefore, diagnostic instruments are needed which circumvent the influence of language skills on predictor variables. Elbro et al. (2012) found that their measure of artificial decoding was able to discriminate dyslexic from non-dyslexic adult second-language learners.

Against this background, the current study aimed to assess whether SSP measured at the young age of 4–5 years predicts reading performance 3 years later. As an auxiliary question, it was tested whether multilingual children differ in SSP performance from monolingual children.

## Materials and Methods

### Sample and Procedures

At the first measurement time point (T1), 56 preschool children (34 female: 17 multilingual, 17 monolingual; 22 male: 12 multilingual and 10 monolingual) took part. All multilingual children had exposure to the German language for at least 2 years. It was ensured that all children understood the instructions. Children were aged between 4.01 and 5.99 (*M* = 5.00; *SD* = 0.50). At T1, children were tested individually in a quiet room of their day-care center. The SSP, letter knowledge (LK), and non-verbal intelligence (IQ) were assessed (Weber et al., 2014).

Three years later, 17 children were retested (10 girls, 11 multilingual). At the time of retesting, the children were in first (*n* = 4), second (*n* = 11), and third (*n* = 2) grades, respectively. The testing took place individually at each child’s home. Reading fluency and reading comprehension were tested. As a further control variable, non-verbal IQ was additionally measured at T2.

This study was conducted in accordance with the Declaration of Helsinki. Informed written consent was obtained from all parents of participants. The study was approved by the Ethics Committee at the Medical Faculty of RWTH Aachen University.

### Instruments

#### T1: Measures in Preschool

##### SSP

**FIGURE 1:**
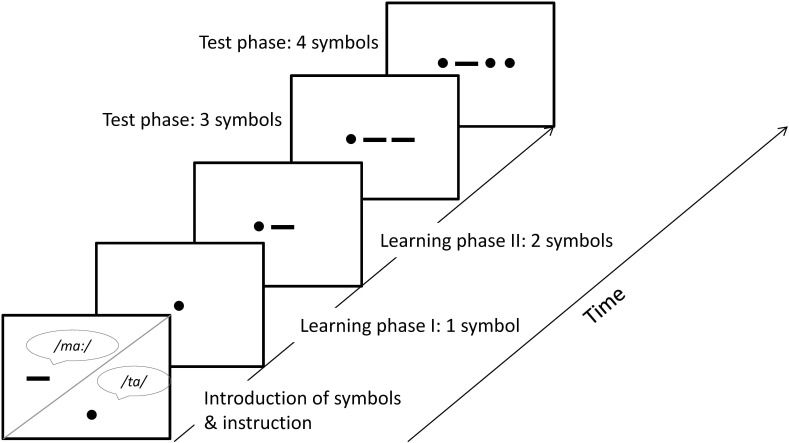
Task procedure of the sound-symbol paradigm (SSP; Horbach et al., 2015).

This task was a computer-based version based on an existing paper–pencil task of Köhn and Voß (unpublished thesis) and was described in Horbach et al. (2015) as follows: the task was designed to simulate the reading process schematically. The first part of the task was a learning phase, similar to a classical PAL-task, where the children learn to associate verbal sounds with graphical symbols. It was followed by a second learning phase. The main part of the task was the test phase which required the serial application of the newly-learned correspondences. To keep the influence of phonological processing and working memory load as low as possible, the children learned only two associations (**Figure 1**).

##### Learning phase 1

The task started with a voice introducing two symbols: a dot “•” and a dash “**—**”. Each symbol was presented separately on the screen and the voice explained that the dot is called /ta/ and the dash is called /ma:/. The children were instructed to name the symbols. Stimuli were presented on a 23-in TFT display in a fixed order. If the child responded correctly, he/she received positive feedback (“yes, this was /ta/”), and the next trial appeared. If the child’s response was incorrect, the experimenter provided negative and corrective feedback (e.g., “no, this was /ma:/”), and the trial was repeated. Due to this repetition, the exposure to both stimuli was individual to each child. The task was performance sensitive in that children only reached the next learning phase after passing through a minimum of 10 correctly-solved trials. Performance was assessed as the percentage of correctly solved trials.

##### Learning phase 2

To prepare the children for the following test phase, they had to name the recently learned symbols in a string of two symbols (e.g., visual stimulus: “• **—**” correct response: “/ta ma:/”). Again, feedback was provided and at least 10 items had to be solved correctly (abort criterion max. 20 trials). Performance was assessed as the percentage of correctly solved trials.

##### Test phase

The test phase required the serial application of the newly-learned correspondences. Twelve trials with three or four symbol strings were presented in the same way as in the learning phase, except that feedback was no longer given (six trials for each string length). The correlation between performance on three and four symbol strings was high (*r* = 0.72). Since all analyses showed the same patterns for three and four symbols, the two scores were combined. The items of the task had high internal consistency (Cronbach’s alpha = 0.87).

##### Non-verbal intelligence measure

Non-verbal IQ was measured using Raven’s Colored Progressive Matrices (CPMs; Bulheller and Häcker, 2002). The CPM is designed to measure the child’s reasoning ability, which is referred to as general IQ.

##### Letter knowledge (LK)

In an individual letter naming task, the children were asked to name all 26 upper case letters of the German alphabet. These were presented in a random order on a white sheet of paper. One point was given for each correctly pronounced letter. Both letter names and letter sounds were possible answers.

#### T2: Measures 3 Years Later

##### Reading fluency

Reading performance was measured using a standardized word reading fluency test, the Salzburg Reading and Spelling Test (SLRT-II; Moll and Landerl, 2010). The SLRT-II test measures reading speed and accuracy of words and pseudowords within a 1 min reading fluency task. The sum of correctly-read words and pseudowords was measured.

##### Reading comprehension

The standardized reading comprehension test ELFE 1-6 (Lenhard and Schneider, 2006) was used to assess reading comprehension on word, sentence, and text level. Word reading comprehension requires the child to decide which word out of four fits best to a given image. Sentence comprehension requires the child to choose one of four words that fits best into a given sentence. On text level, small stories had to be read and questions had to be answered. The cumulated z-score of all three subtests was used to score reading performance.

##### Non-verbal intelligence measure

Non-verbal IQ was measured with the short version of CFT1-R (Weiß and Osterland, 2013).

## Results

**Table 1** shows the performance in predictor measures assessed at T1. High dropout from T1 to T2 occurred for several reasons (participants moved, declined participation, or could not be contacted). Thirty percent of the participants could be retested. In order to determine whether the reduced sample at T2 significantly differed from the full sample at T1, one-sample *t*-tests were computed for SSP, LK, IQ, and age at T1 with the respective mean of each variable at T1 as test value. The reduced sample assessed at T2 did not differ in its performance in the predictor measures SSP, LK, and IQ (**Table 1**) from the reference values. Chi-squared tests revealed that the distribution of sex was nearly equal, with 39% boys at T1 and 41% boys at T2 [*χ^2^*(1) = 0.03, *p* = 0.854]. At T2, also the proportion of multilingual children was comparable to T1 [*χ^2^*(1) = 1.90, *p* = 0.168]. These results suggest that the group of children participating at T2 was a random sample from the initial group at T1 and, as a consequence, that there was no systematic dropout between T1 and T2.

**Table 1:** Comparison between the reduced sample (T2) at T1 with the full sample (T1).

	T1 (*n* = 56) *M* (*SD*)	T2 (*n* = 17) *M* (*SD*)	*t*	*df*	*p*
Age	5.00 (0.50)	5.10 (0.48)	0.87	16	0.397
SSP learn 1 in %	77.88 (13.08)	82.28 (12.70)	1.43	16	0.172
SSP learn 2 in %	68.62 (13.71)	67.88 (15.19)	−0.200	16	0.844
SSP Test in %	36.01 (30.41)	47.55 (33.30)	1.43	16	0.172
LK max. 26	3.77 (5.87)	5.47 (6.10)	1.16	16	0.264
IQ	87.25 (14.60)	91.35 (17.10)	1.82	16	0.087

*SSP, sound-symbol paradigm; LK, letter knowledge.*

### Performance on Predictor Measures at T1

In the first learning phase of SSP, the children responded accurately nearly 80% of the time. In the second learning phase, 70% of the response was accurate. In the test phase where complexity of the task grew and feedback was no longer given, the children responded with an average of 30–40% accuracy.

Concerning LK floor effects were observed. On average, the children of this young age were only able to identify 3.77 letters out of 26.

Forty percent of the children reached an IQ value below average. This was not unexpected, given the fact that children were recruited in regions with relatively low socioeconomic status. It was assured that all children understood instructions.

**FIGURE 2:**
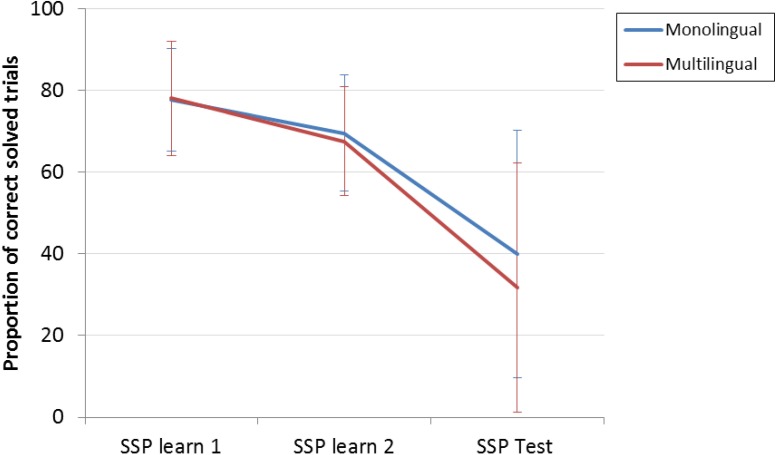
SSP performance of multilingual and monolingual children.

In order to compare the performance of monolingual and multilingual children on the different levels of the SSP a two-way repeated measures ANOVA (Greenhouse-Geisser corrected) was performed, with task level as within-subject factor and group as between-subject factor. A significant main effect of task level, *F*(2.10,113.37) = 85.84, *p* < 0.001 was observable. This showed that across groups, performance decreased as task complexity increased (**Figure 2**). No significant main effect of group [*F*(1,54) = 0.71, *p* = 0.402] indicated that multilingual and monolingual children in general performed similarly.

### Prediction of Reading Performance 3 Years Later

Monolingual and multilingual children did not differ in their performance of SSP at T1, and group sizes were small. Therefore, the longitudinal prediction analyses were performed over the total sample.

Sound-symbol paradigm measured at T1 was a strong correlate of reading performance 3 years later (word reading fluency *r* = 0.86, *p* < 0.001; pseudoword reading fluency *r* = 0.81, *p* < 0.001; reading comprehension *r* = 0.82, *p* < 0.001). LK did not correlate significantly with 3 year later reading, presumably due to floor effects of LK. IQ (T1) was a moderate correlate of word fluency (*r* = 0.60, *p* = 0.011) and pseudoword fluency (*r* = 0.54, *p* = 0.030) measured at T2. For all correlations, see **Table 2**.

**Table 2:** Pearson correlations between predictor measures (T1) and outcome measures (T2).

		1	2	3	4	5	6	7
T1	1. SSP	–		.				
	2. LK	0.179	–					
	3. IQ	0.609^∗∗^	0.007	–				
	4. Age	0.340^∗^	0.286^∗^	0.429^∗∗^	–			
T2	5. Word fluency	0.855^∗∗^	0.043	0.602^∗^	0.282	–		
	6. Pseudoword fluency	0.807^∗∗^	−0.033	0.543^∗^	0.150	0.914^∗∗^	–	
	7. Comprehension	0.817^∗∗^	0.170	0.463	−0.303	0.686^∗∗^	0.747^∗∗^	
	8. IQ	0.628^∗∗^	0.289	0.649^∗∗^	0.208	0.641^∗∗^	0.610^∗^	0.574^∗^

*SSP, sound-symbol paradigm; LK, letter knowledge; word fluency, word reading fluency (SLRT II); pseudoword fluency, pseudoword reading fluency (SLRT II); comprehension, reading comprehension (ELFE 1-6); p^∗^ < 0.05, p^∗∗^ < 0.01.*

**FIGURE 3:**
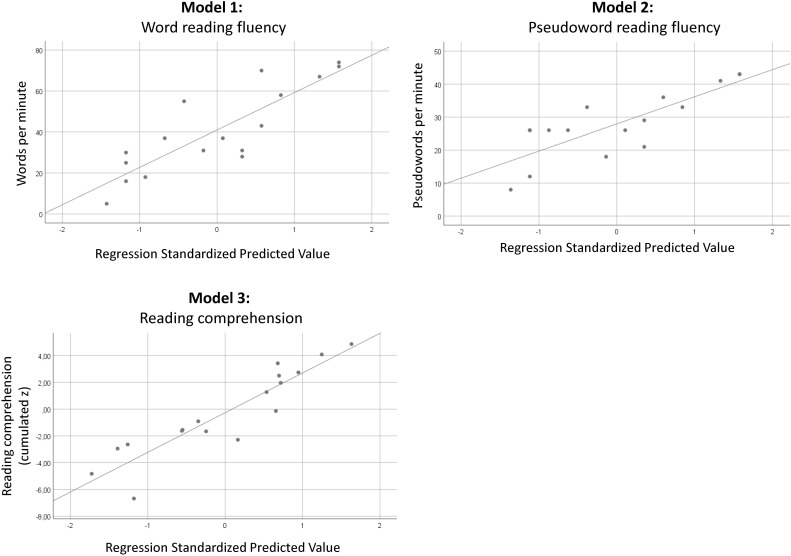
Scatterplots of the best prediction models for each reading outcome variable.

Linear regression models with SSP, IQ, LK, and age as predictors were computed for each reading outcome variable (**Table 3**). Applying a threshold *p*-value of 0.10, non-significant predictors were removed in order to find the best model in terms of fit and parsimony for each variable. Adjusted *R*^2^ was used as a method of cross-validation. Scatterplots of the final models are shown in **Figure 3**.

**Table 3:** Final linear regression models.

Model	Dependent variable	Predictors	*B*	*SE B*	*β*	Adj. *R*^2^
1	Word fluency					0.72
		Constant	14.96	4.94		
		SSP	0.55	0.09	0.86^∗∗∗^	
2	Pseudoword fluency					0.63
		Constant	16.73	2.69		
		SSP	0.24	0.05	0.81^∗∗∗^	
3	Comprehension					0.82
		Constant	10.16	3.74		
		Age	−2.84	0.74	−0.42^∗∗^	
		SSP	0.09	0.01	0.87^∗∗∗^	

*Predictor variables included in each initial model were age, letter knowledge, IQ, and sound-symbol paradigm (SSP); p^∗∗^ < 0.01, p^∗∗∗^ < 0.001.*

Model 1: For word reading fluency as dependent variable, SSP was the only significant predictor and explained a variance of 71% [*F*(1,15) = 40.647, *p* < 0.001]. IQ, LK, and age did not contribute to the final model.

Model 2: The analysis with pseudoword fluency as a dependent variable revealed the same pattern. Again, SSP was the unique significant predictor. The explained variance was 63% [*F*(1,14) = 26.091, *p* < 0.001].

Model 3: In the third analysis, SSP and age contributed significantly to the variance of reading comprehension. Eighty-two percent of the variance in reading comprehension is explained by the model [*F*(2,14) = 36.462, *p* < 0.001].

In order to show the robustness of the models, a second way of cross-validation was applied. The bivariate Pearson coefficients of the correlations were compared between the predicted value and the dependent variable of a randomly selected 60% subsample with a 40% subsample. For the first model with the dependent variable “word reading,” the correlation of 60% subsample is *r* = 0.878, *p* < 0.001 and of the 40% subsample *r* = 0.870, *p* = 0.024. Similar patterns are found for the second and third models with pseudoword reading and reading comprehension as dependent variables. For pseudoword reading, the correlations were *r* = 0.805, *p* = 0.005 and *r* = 0.893, *p* = 0.017. For reading comprehension, the correlations were *r* = 0.962, *p* = 0.002 and *r* = 0.910, *p* < 0.001. The high and nearly equal correlations suggest that the models are robust.

In order to find out whether the prediction of SSP is specific to reading or unspecific, i.e., as well predictive for general cognitive abilities, a further regression analysis was conducted. IQ measured at T2 (IQ_T2_) served as dependent variable and SSP was included as predictor. SSP explained with 35% a significant albeit smaller amount of variance in non-verbal IQ_T2_ (β = 0.63, *p* = 0.007*)* as in reading variables.

## Discussion

This longitudinal study aimed to determine the predictive value of a Morse-code like SSP assessed in preliterate preschool children, aged 4–5, of reading performance 3 years later.

The results showed that SSP substantially predicted reading fluency and reading comprehension 3 years later. For reading fluency measures, the influence of further predictor variables (age, IQ, and LK) was not significant and SSP served as a sole predictor. Reading comprehension was best explained by SSP and age. The finding that SSP contributed considerably lower to the variance of non-verbal IQ_T2_ as to the variance of reading is consistent with a specific prediction effect on reading. The amount of variance SSP explained in reading 3 years later was remarkably high, with an explained variance between 63 and 82%, depending on the outcome reading variable. We suggest this prediction is that accurate because SSP challenges students to acquire completely new sound-symbol relations, which simulates a core aspect of natural reading acquisition. Good or poor performance of SSP may result from stable or instable association of sound-symbol pairs in the learning part of SSP. This corresponds to the hypotheses of Blomert and Willems (2010) that letter speech sound binding plays a causal role in learning to read. In line with this hypotheses and our findings, Karipidis et al. (2018) showed an artificial letter training predicts reading. Furthermore, they demonstrated that neural underpinnings are significantly related to later reading performance.

Previous studies that used paradigms comparable to SSP in preliterate children found smaller effect sizes; however, additional predictor variables were used and reading was predicted only 1 year later (Horbach et al., 2015: *R*^2^ = 0.36, Gellert and Elbro, 2017: *R*^2^ = 0.55). There was also a stronger correlation between SSP and reading performance observed (*r* = 0.80 to *r* = 0.86) compared to Horbach et al. (2015), using the same paradigm *(r* = 0.36). A possible explanation is that SSP works especially well with the currently addressed age group of 4–5-year-old children. The paradigm is designed to be learned easily, because the children have to learn only two sound-symbol associations. After the learning phase, they simply have to string the sound of the displayed symbols together. They are not required to blend phonemes into another as it is required in the paradigm of Gellert and Elbro (2017). Maybe these low demands make the paradigm especially useful for *young* children.

SSP’s ability to predict later reading is partly due to its dynamic nature. A dynamic test aims to measure a child’s potential to learn, in contrast to static assessments (e.g., PA, RAN, and LK), which measure the current attainment of the child (Lidz, 1983, 1996). Also previous studies demonstrated the superiority of dynamic measures in comparison to static assessments in the prediction of reading (Petersen et al., 2016; Gellert and Elbro, 2017, 2018). As reading acquisition is a learning process, it seems obvious that paradigms which include the learning aspect can explain an extra amount of variance in reading additionally to a specific cognitive demand of the predictor measure. Furthermore, a dynamic measure avoids the problem of the influence of environmental support, which is always a limitation of static measures (Petersen et al., 2016).

In the current study, the static assessment of LK did not contribute to the explained variance of reading, although it is regarded as one of the strongest predictors of reading before formal reading instruction starts (Scarborough, 1998; Hammill, 2004). We assume that children in our socioeconomically diverse sample did not have much opportunity to acquire LK in their home environment. But this deficit does not implicate a disorder in later reading. Children who have limited literacy experience due to weak socioeconomically background are at risk of being overdiagnosed with a learning disability (Artiles et al., 2002). Also, LK may play a more important role in older children. Most studies that identify LK as important predictor assessed older children in their last kindergarten year, i.e., children are aged six on average. The 4- and 5-year-old children of the current sample were rarely familiar with letters. Therefore, floor effects could also have led to a poor predictive value in our study. The problem of floor effects in early pre-reading measures is well known (Catts et al., 2008). The advantage of SSP is that children are learning the associations directly in the test situation, so it is independent from pre-knowledge, age, or educational support. In line with the findings of Gellert and Elbro (2017), we conclude that the learning aspect of SSP is an essential part in the task and, therefore, leads to the strong predictive value of reading.

A further question of the study was whether SSP is appropriate for multilingual children. In many of Germany’s day-care centers, children of various origins grow up together. A method that is equally suitable for monolingual and multilingual children allows a fair, language-independent assessment. Second-language learner often shows linguistic delays compared to monolingual children (Schwippert et al., 2008). They have fewer opportunities to build up sufficient language skills in the environment language compared to monolingual children. It is, therefore, not surprising that children with migration background scored significantly lower in the language dependent measures PA and RAN than monolingual children (Weber et al., 2007). It was also found that PA did not contribute to the prediction of reading in second-language learners, whereas it was the strongest predictor in monolingual children (Duzy et al., 2013). Hence, it is unclear whether these language-dependent abilities predict reading in multilingual children as reliable as in monolingual children or the use of those language-dependent predictors leads to false risk diagnoses (for an overview, see Cline and Shamsi, 2000). This problem could be avoided by using the language-independent measures like the SSP task. Elbro et al. (2012) showed in adult second-language learners that the performance in their dynamic measure of decoding was able to differentiate dyslexic from non-dyslexic readers. In this line, the current study demonstrated that SSP performance of monolingual and multilingual children was comparable. No differences were detected in learning the new sound-symbol pairs or in serial processing. Thus, the language independent nature of the task makes it as appropriate for multilingual children.

### Limitations of the Current Study

The high dropout after 3 years at T2 led to a small sample size. The comparison of the reduced sample at T2 with the full sample at T1 showed no significant differences in any measured characteristics. Thus, it is reasonable to assume that no differential attrition took place. Nevertheless, generalization of the results should be avoided. Although substantial effects of the prediction analyses can be observed even though the sample size is relatively small, in further studies with bigger sample sizes, the predictive value of SSP in monolingual and multilingual children should be differentiated.

The common predictor measures PA and RAN were not used at T1 in order to avoid the problem that multilingual children are confronted with language-dependent measures. Beside this, no control condition has been implemented. Therefore, this study cannot speak to the specificity of SSP. Previous studies showed that comparable paradigms share variance with PA and RAN but also contribute uniquely to the variance of reading performance (Horbach et al., 2015; Gellert and Elbro, 2017). The overall explained variance of reading in previous studies being smaller, although more predictor measures were included, suggests the specific part of the explained variance contributed by SSP is relatively high.

## Conclusion

The present study extended the findings of current literature that SSPs can predict reading to the young age group of 4–5-year-old preschoolers. Future work will test this paradigm in less transparent languages like English and explore its potential as a future assessment in the study of early reading development.

## Author Contributions

JH, TG, and SH designed the study. FO and KW performed the experiments and recruited the participants. JH and FO performed the calculations. WS helped to analyze the data. JH wrote the manuscript. RR, TG, and SH supervised the study. All authors provided critical feedback and commented on the manuscript.

## Conflict of Interest Statement

The authors declare that the research was conducted in the absence of any commercial or financial relationships that could be construed as a potential conflict of interest.
